# MicroRNA-221 is a potential biomarker of myocardial hypertrophy and fibrosis in hypertrophic obstructive cardiomyopathy

**DOI:** 10.1042/BSR20191234

**Published:** 2020-01-10

**Authors:** Derong Huang, Zhongxiu Chen, Jie Wang, Yucheng Chen, Daxing Liu, Ke Lin

**Affiliations:** 1 Department of Cardiovascular Surgery, West China Hospital, Sichuan University, Chengdu, Sichuan 610041, China; 2 Department of Cardiology, West China Hospital, Sichuan University, Chengdu, Sichuan 610041, China; 3 Department of Cardiovascular Surgery, Affiliated Hospital of Zunyi Medical University, Zunyi, Guizhou 563100, China

**Keywords:** hypertrophic obstructive cardiomyopathy, MicroRNA, myocardial fibrosis, myocardial hypertrophy

## Abstract

Aim: Circulating microRNA expression has become a biomarker of cardiovascular disease; however, the association of microRNA expression between circulation and myocardium in hypertrophic cardiomyopathy remains unclear. The present study aimed to find a circulating biomarker correlated not only to myocardial expression, but also to cardiac hypertrophy and fibrosis. Method: Forty-two cases of hypertrophic obstructive cardiomyopathy (HOCM) diagnosed by echocardiography and magnetic resonance were analysed for microRNA expression in plasma and myocardial tissue. Results: The results showed that myocardial miR-221 was significantly increased (*z* = −2.249, *P* = 0.024) and significantly correlated with collagen volume fraction (CVF) (*r* = 0.516, *P* < 0.001), late gadolinium enhancement (LGE) (*r* = 0.307, *P* = 0.048), and peripheral circulation (*r* = 0.434, *P* = 0.004). Moreover, circulating miR-221 expression was significantly correlated with CVF (*r* = 0.454, *P* = 0.002), LGE (*r* = 0.630, *P* = 0.004), maximum interventricular septal thickness (MIVST) of echocardiography (*r* = 0.318, *P* = 0.042), and MIVST of magnetic resonance (*r* = 0.342, *P* = 0.027). The area under the receiver operating characteristic curve of miR-221 was 0.764. Conclusions: Circulating miR-221 is consistent with that in myocardial tissue, and correlated with myocardial fibrosis and hypertrophy. It can be used as a biomarker for evaluating myocardial hypertrophy and fibrosis in HOCM.

## Introduction

Hypertrophic obstructive cardiomyopathy (HOCM) is a common hereditary heart disease, characterised by myocardial hypertrophy, dysplasia and fibrosis. Persistent hypertrophy of cardiomyocytes is a key risk factor for the development of heart failure [1], and myocardial fibrosis is a powerful driving factor for ventricular remodelling and heart failure [2,3]. MicroRNAs (miRs) are considered to be involved in many physiological and pathological processes, including cardiomyocyte proliferation [4], differentiation [5], apoptosis [6], fibrosis, and hypertrophy [4,7]. Circulating miR has recently been found to be a biological marker of various heart diseases (e.g. miR-208a is a biomarker of acute myocardial infarction in humans [8], miR-19b of heart failure [9], miR-126 and miR-199 of coronary heart disease [10], and miR-499 of hypertension [11]). In addition, circulating miR, such as mir-29a, was also found to be a biological marker of hypertrophy and fibrosis in hypertrophic cardiomyopathy (HCM) [7,12]. However, miRs in peripheral circulation may come from other involved organs (such as the aortic arch [7]). None of the above studies was controlled by myocardial tissue. Hence, the present study aimed to find a circulatory miR not only associated with myocardial expression, but also with clinical phenotype of HOCM patients.

## Materials and methods

### Patients

All subjects gave written informed consent to participate in the study. The research protocol was approved by the Ethics Committee of the West China Hospital of Sichuan University (Sichuan, China) (2018250), and the study conformed to the principles of the Declaration of Helsinki.

The present study consisted of 42 consecutive patients with HOCM who underwent transaortic extended septal myectomy between January 2014 and June 2018 at West China Hospital. The diagnosis of HOCM was based on the presence of a hypertrophied myocardial wall (usually ≥15 mm in adults or the equivalent relative to body surface area in children), as identified by echocardiography, and the absence of other cardiac or systemic diseases that cause hypertrophy, in accordance with the European Society of Cardiology (ESC) Guidelines on diagnosis and management of HOCM [13]. The indications for surgical myectomy were: (1) severe symptoms or syncope or near-syncope despite optimal medical therapy, (2) a left ventricular outflow tract gradient ≥ 50 mm Hg at rest or with provocation by transthoracic echocardiography, (3) no suitable target vessel for septal alcohol ablation, and (4) patient voluntary acceptance. Their myocardial tissue and peripheral blood were collected, blood samples were collected before cardiopulmonary bypass began, intraoperative excision of myocardial tissue was divided into two parts: one was stored in liquid nitrogen immediately for miRNA detection and the other was fixed in 4°C formalin for histopathological analysis.

Blood samples of 30 healthy volunteers (from July 2017 to August 2018) matched for age and sex, with normal physical examination and no systemic disease, hypertension, and HOCM family history were collected. Then, eight myocardium specimens from patients who died from non-cardiovascular diseases were used as controls (from West China Hospital of Sichuan University and Zunyi Medical University Affiliated Hospital).

### Echocardiography

Echocardiography was performed by IE33 ultrasound system (Philips Medical Systems, Andover, MA, U.S.A.) equipped with a S5-1 transducer (frequency 1.7–3.4 MHz), according to American Society of Echocardiography guidelines [14]. Interventricular septal thickness, left ventricular diastolic diameter, and left ventricular posterior wall thickness were measured in the parasternal long-axis views at end diastole. Three consecutive measurements were conducted to find the average value. Left ventricular mass (LVM) was calculated by the Devereux correction formula: LVM (g) = 0.8 × 10.4 × [(IVSt + LVPWt + LVDd)^3^ − LVDd^3^] + 0.6. Then, LVM divided by body surface area equals the LVM index (g/m^2^), left ventricular outflow tract gradient and ejection fraction were calculated as our previously reported [15].

### CMR

CMR was carried out using 3.0-T magnetic resonance imaging system (Gyroscan Intera, Philips Software System, The Netherlands). In the continuous images of the left ventricular short axis, the largest was the end diastolic image, and the smallest was the end systolic image. The left ventricular wall and interventricular septal thickness were measured. Through the post-processing analysis software (NUTS2013) we could obtain the left ventricular ejection fraction, left ventricular end-diastolic volume, left ventricular end-systolic volume, and left ventricular mass and then standardised treatment with body surface area. The corresponding index was also obtained. LGE was performed with phase-sensitive inversion sequence. The left ventricular short-axis and long-axis images were collected 10 min after injection of contrast agent (Gd-DTPA, 0.2 mmol/kg; flow rate, 3.5 ml/s). Left ventricular myocardial segmentations were performed according to the American Heart Association standard [13]. The degree of late gadolinium enhancement (LGE) was quantitatively analysed using the general visual integration method.

### Assessment of myocardial fibrosis

Samples were fixed in 4% paraformaldehyde for 48 h, embedded in paraffin, sectioned at 5 µM, and stained with Masson’s trichrome. Images were obtained using a NanoZoomer 2.0 HT pathology slice scanner (C9600-12, Japan) with 40× scanning resolution for analysis. To evaluate the myocardial fibrosis degree, collagen volume fraction (CVF) was analysed using Image-Pro PLUS software (Media Cybernetics, Silver Spring, MD, U.S.A.) by an experienced pathologist blinded to the clinical data. For each cardiac sample, a total of 10 fields were analysed, and CVF was expressed as the mean percentage of Masson’s trichrome-stained area per total myocardial tissue area. Each slice was calculated by two independent observers, and the error between the two values is less than 3%, and their average value was the CVF of the sample.

### Myocardial miRNA analysis

The myocardial tissue was taken out from the liquid nitrogen and powderised. Total RNA was isolated with TRIzol reagent (Life, U.S.A.) according to manufacturer’s instructions. Ultramicro nucleic acid protein analyser (Scandrop100, Germany) was used to determine RNA concentration and purity. Reverse transcription kit (TUREscript 1st-Strand cDNA SYNTHESIS Kit, Aidlab, U.S.A.) was used to perform reverse transcription operation with EASYCYCLER 96 PCR Instrument (Analytik Jena, Germany). Quantitative real-time polymerase chain reactions (PCR) were completed by QTOWER 2.2 fluorescence quantitative PCR instrument (Analytik Jena, Germany). The reaction conditions are as follows: 95°C for 10 min followed by 40 cycles of 95°C for 2 s, 65°C for 20 s, and 70°C for 10 s. Then, the melting curve was analysed at 60–95°C to determine the final product. There were triplicates in each sample, and the difference between them was <0.25. Analytik Jena qPCRsoft 3.2 was used for data analysis. The *C*_T_ value was defined as the number of cycles in which the fluorescence signal passes through the threshold line. Each miR expression was standardised using a stably expressed housekeeper gene U6. The relative expression was calculated using 2^−∆∆Ct^ method. A total of 11 miRs previously associated with the fibrosis of heart, liver or kidney were selected for investigation: miR-9, miR-31, miR-33, miR-93, miR-15, miR-21, miR-19b, miR-221, miR-222, miR-433, and miR-155. All primers in the present study were purchased from Rib Bio Biotech Co., Ltd. (Guangzhou, China). We screened their differential expression in myocardial tissue and selected interested miRs to further explored their correlation with peripheral circulation and hypertrophic phenotype.

### Blood collection and plasma separation

The patient’s antecubital vein blood (approximately 3–5 ml) was collected before operation, placed in a vessel containing ethylenediaminetetraacetic acid, anticoagulants, immediately placed in a 4°C refrigerator, then centrifuged for 10 min (4°C, 1000 ***g***), added with supernatant liquid, and stored in liquid nitrogen. The control group was treated in the same way.

### MiRNA detection in plasma

Total RNAs in plasma were extracted using QIAGEN sample and assay technologies kit (QIAGEN, Germany), according to purification of total RNA, including miR from the serum and plasma protocol. During extraction, cel-miR-39 was added as an external parameter, and each miR expression was standardised by quantitative addition of exogenous cel-miR-39. Reverse transcription and PCR reaction were identical with the tissue detection method. Two of the 11 selected miRs, myocardial miR-19b and miR-221, significantly correlated with the degree of cardiac fibrosis. Thus, they were furtherly detected in circulation.

### Statistical analysis

Statistical analysis was performed using SPSS version 22.0 (IBM, Armonk, NY). Categorical data were summarised as numbers and proportions (percentage), whereas continuous data were summarised as mean ± standard deviation or median. Kolmogorov–Smirnov test was applied to verify the data that conform to normal distribution. If it is a normal distribution, independent-samples *t*-test was used; otherwise, Wilcoxon test was used. Chi-squared test was used for discrete variables. Spearman’s rank correlation was used to analyse the correlation between two variables. Receiver-operating characteristic (ROC) curve was carried out to calculate the area under curve (AUC) to assess diagnostic value. All *P* values < 0.05 were considered statistically significant.

## Results

### Patient demographics

A total of 42 patients with HOCM (29 men and 13 women) were included in the present study. The average age was 46.24 ± 15.48 (range 15–69) years. The proportion of resting and provocating obstruction type was 66.67% (28/42) and 33.33% (14/42), respectively. All patients were treated with surgical operation to relieve obstruction. Patient’s clinical features, echocardiography, and CMR parameters are shown in Tables 1 and 2.

**Table 1:** Clinical, echocardiographic, and CMR characteristics in patients with HOCM and control group (blood)

Group (*n* = 30)	HOCM patients (*n* = 42)	Control (*n* = 30)	*P* value
Age, yrs	46.24 ± 15.48	45.57 ± 9.75	0.835
Male, *n* (%)	29(69.05)	20(66.67)	0.598
Heart rate, bpm	73.95 ± 12.87	72.77 ± 9.51	0.670
Systolic blood pressure, mmHg	116.52 ± 18.27	112.33 ± 12.87	0.285
Diastolic blood pressure, mmHg	70.26 ± 12.36	67.77 ± 9.41	0.356
Family history of HCM, *n* (%)	8(19.05)	0	0.011
Family history of sudden death, *n* (%)	3(7.14)	0	<0.001
Syncope, *n* (%)	7(16.67)	0	0.020
Paroxysmal ventricular tachycardia, *n* (%)	5(11.90)	0	<0.001
Atrial fibrillation, *n* (%)	4(9.52)	0	0.082
**Complicated disease**			
Absent, *n* (%)	35(83.33)	30(100)	0.019
Hypertension, *n* (%)	5(11.90)	0	<0.001
Diabetes, *n* (%)	2(4.76)	0	0.220
**Pharmacological therapy**			
No therapy, *n* (%)	3(7.14)	30(100)	<0.001
ACEI/ARBs, *n* (%)	5(11.90)	0	0.050
β-Blockers, *n* (%)	22(52.38)	0	<0.001
Calcium antagonists, *n* (%)	1(2.38)	0	0.395
Diuretics, *n* (%)	13(30.95)	0	0.001
**Echocardiogram**			
Left ventricular outflow tract gradient, mmHg	60.61 ± 28.97	/	/
SAM phenomenon, *n* (%)	35(83.33)	0	0.019
Interventricular septal thicknes, mm	23.19 ± 5.82	/	/
Ejection fraction of left ventricle, (%)	70.09 ± 11.21	/	/
**Mitral regurgitation**			
Absent, *n* (%)	17(40.48)	30(100)	<0.001
Mild, *n* (%)	14(33.33)	0	<0.001
Moderate, *n* (%)	10(23.81)	0	0.004
Severe, *n* (%)	1(2.38)	0	0.395
**Cardiac magnetic resonance imaging**			
Interventricular septal thickness, mm	25.38 ± 7.24	/	/
Ejection fraction of left ventricle, %	63.79 ± 11.43	/	/
			

Abbreviations: ACEI, angiotensin-converting enzyme inhibitors; ARB, angiotensin II type 1 receptor blockers; HOCM, hypertrophic obstructive cardiomyopathy patients. Values are expressed as mean ± SEM, and number or percentage of patients. *P* value versus control group.

**Table 2:** Clinical characteristics in HOCM patients and control group (myocardium)

Group characteristics	HOCM patients (*n* = 42)	Control (*n* = 8)	*P* value
Age, yrs	46.24 ± 15.48	46.13 ± 14.48	0.985
Male, *n* (%)	29(69.05)	5(62.50)	0.319
Heart rate, bpm	73.95 ± 12.87	72.77 ± 9.51	0.670
Systolic blood pressure, mmHg	116.52 ± 18.27	117.63 ± 11.40	0.871
Diastolic blood pressure, mmHg	70.26 ± 12.36	68.38 ± 8.02	0.681
Family history of HCM, *n* (%)	8(19.05)	0	0.178
Family history of sudden death, *n* (%)	3(7.14)	0	0.044
Syncope, *n* (%)	7(16.67)	0	0.213
Paroxysmal ventricular tachycardia, *n* (%)	5(11.90)	0	0.304
Atrial fibrillation, *n* (%)	4(9.52)	0	0.363
Complicated disease absent, *n* (%)	35(83.33)	8(100)	0.213
Hypertension, *n* (%)	5(11.90)	0	0.304
Diabetes, *n* (%)	2(4.76)	0	0.529
**Pharmacological therapy**			
No therapy, *n* (%)	3(7.14)	8(100)	*<0.001*
ACEI/ARBs, *n* (%)	5(11.90)	0	*0.304*
β-Blockers, *n* (%)	22(52.38)	0	*0.006*
Calcium antagonists, *n* (%)	1(2.38)	0	*0.659*
Diuretics, *n* (%)	13(30.95)	0	*0.113*

Abbreviations: ACEI, angiotensin-converting enzyme inhibitors; ARB, angiotensin II type 1 receptor blockers; HOCM, hypertrophic obstructive cardiomyopathy patients. Values are expressed as mean ± SEM, and number or percentage of patients. *P* value versus control group.

### miR-19b was decreased and miR-221 was increased in myocardium of patients with HOCM

**Figure 1:**
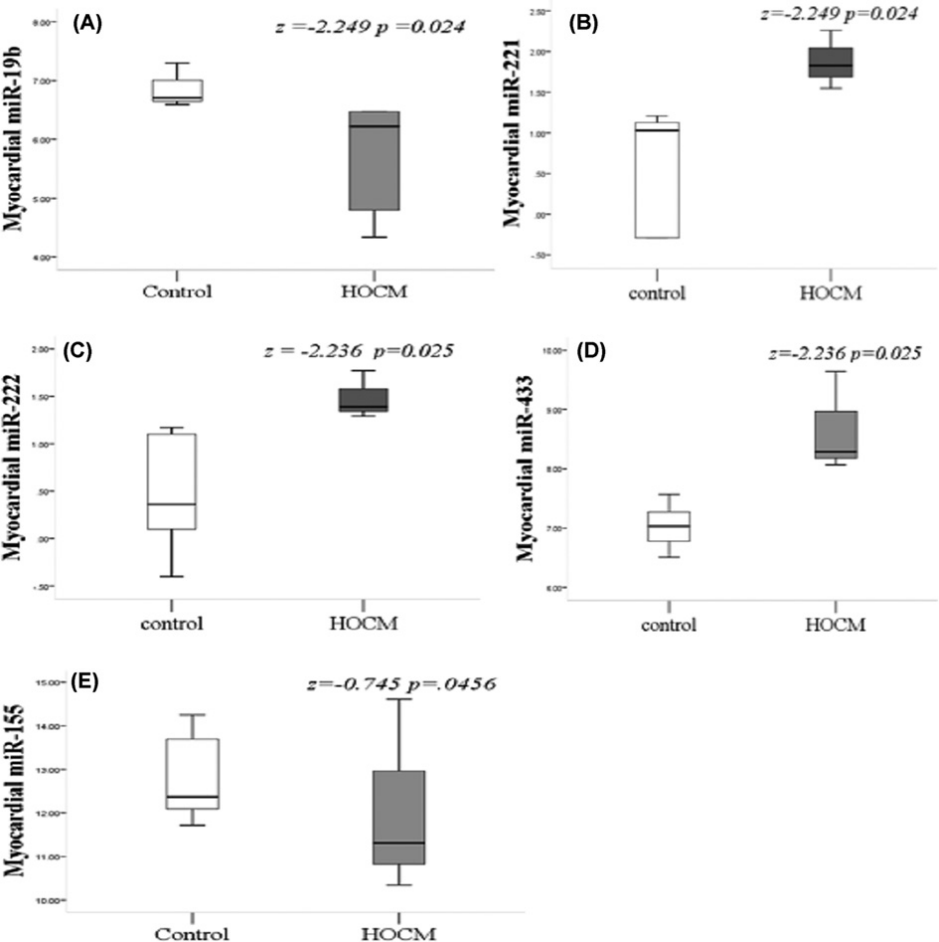
Five myocardial miRNA expressions in HOCM group were significantly abnormal compared with the control group The expression of miR-19b (**A**) and miR-155 (i) were decreased significantly. The expression of miR-221 (**B**), miR-222 (**C**), and miR-433 (**D**) were increased significantly; HOCM, hypertrophic obstructive cardiomyopathy.

Compared with the control group, a total of 11 miRs were included, of which five had significant differences in the HOCM group (Figure 1). The expression of miR-19b (*P* = 0.024) (Figure 1A) and miR-155 (*P* = 0.046) (Figure 1I) were significantly decreased, and three miRNA expression levels were found significantly increased, namely, miR-221 (*P* = 0.024) (Figure 1B), miR-222 (*P* = 0.025) (Figure 1C), and miR-433 (*P* = 0.025) (Figure 1D). There was no significant difference in the expression of the remaining seven miRNAs between the two groups (Supplementary Figure S1 online).

### Myocardial miR-221 and miR-19b correlated with cardiac fibrosis

**Figure 2:**
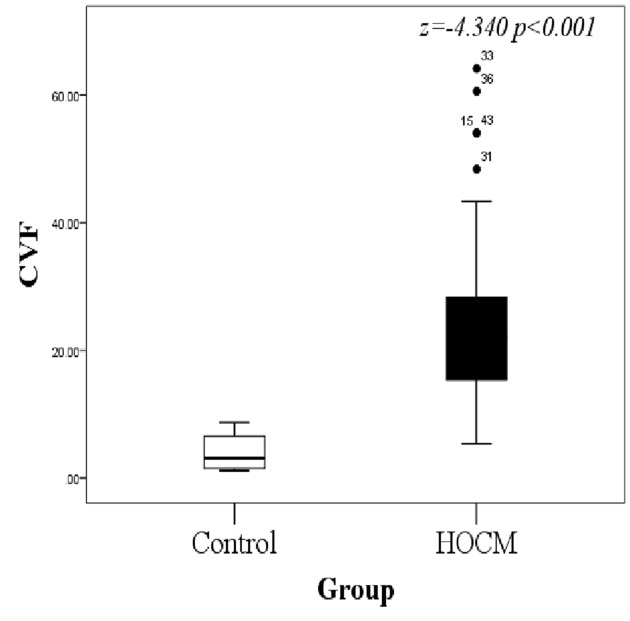
The difference in CVF between HOCM and control groups The control group is significantly lower than the HOCM group; HOCM, hypertrophic obstructive cardiomyopathy; CVF, collagen volume fraction.

**Figure 3:**
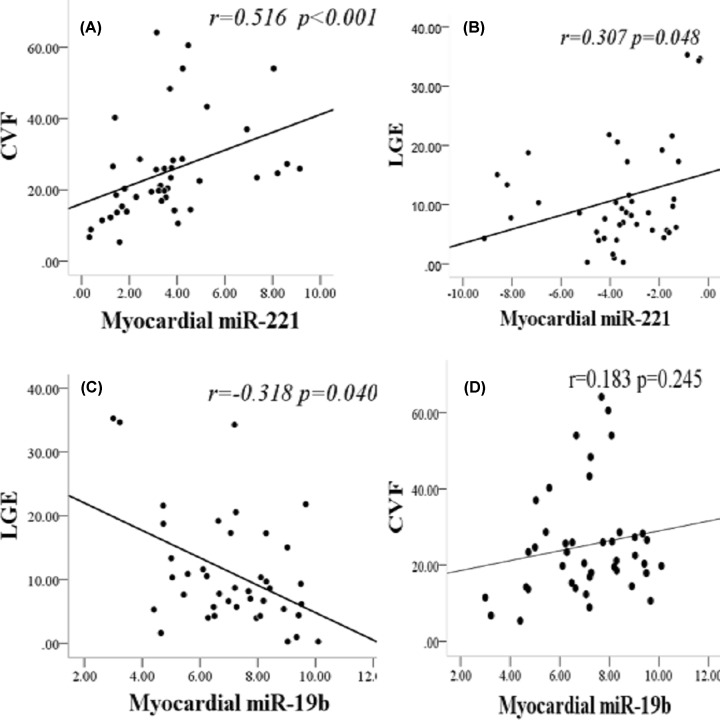
Correlation between myocardial miRNA and myocardial fibrosis (**A**) Myocardial miR-221 was positively correlated with CVF (*r* = 0.516, *P* < 0.001). (**B**) Myocardial miR-221 was significantly and positively correlated with LGE (*r* = 0.307, *P* = 0.048). (**C**) Myocardial miR-19b was negatively correlated with LGE (*r* = −0.318, *P* = 0. 040). (**D**) There was no significant correlation between miR-19b and CVF (*r* = 0.183, *P* = 0.245). LGE means late gadolinium enhancement. *P* values were for bivariate correlation analysis.

The average CVF of the control group and HOCM group was (4.040 ±1.077) and (25.208 ±2.189), respectively, showing significant difference (*P* < 0.001) (Figure 2). Analysis of the correlation between five differentially expressed miRs and the degree of myocardial fibrosis (Figure 3): the expression level of miR-221 was significantly and positively correlated with CVF (*r* = 0.516, *P* < 0.001) (Figure 3A) and late gadolinium enhancement (LGE) (*r* = 0.307, *P* = 0.048) (Figure 3B). MiR-19b expression was inversely correlated with LGE (*r* = −0.318, *P* = 0.040) (Figure 3C). There was no significant correlation between the expression of miR-19b and CVF (*r* = 0.183, *P* = 0.245) (Figure 3D). The remaining miR-222, miR-155, and miR-433 had no significant correlation with LGE and CVF, so they’re excluded from the next step.

### Circulating miR-221 significantly correlated with the clinical phenotype of HOCM

**Figure 4:**
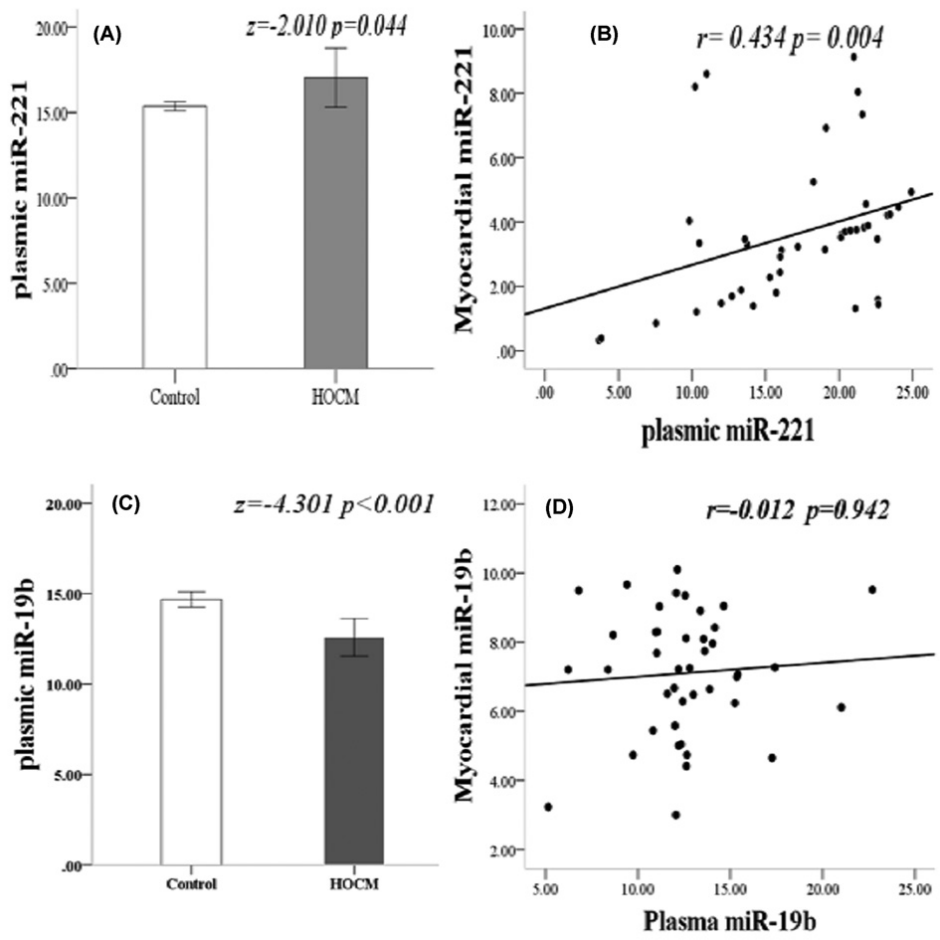
Circulating and myocardial miRNA expression (**A**) MiR-221 expression in plasma was significantly increased (*z* = −2.010, *P* = 0.044). (**B**) There was a positive correlation between miR-221 expression in the plasma and myocardium (*r* = 0.434, *P* = 0.004). (**C**) MiR-19b expression in plasma decreased significantly (*z* = −4.301, *P* ≤ 0.001). (**D**) There was no significant difference in the expression of miR-19b in plasma and myocardium (*r* = −0.012, *P* = 0.942); HOCM, hypertrophic obstructive cardiomyopathy.

Compared with 30 healthy controls, there was a significant difference between miR-221 and miR-19b expression in plasma (Figure 4). The circulating expression of miR-221 in HOCM patients was significantly increased (*P* = 0.044) (Figure 4A) and was positively correlated with myocardial expression level (*r* = 0.434, *P* = 0.004) (Figure 4B). Circulating miR-19b expression was significantly decreased in HOCM patients (*P* ≤ 0.001) (Figure 4C), but had no significant correlation with myocardial expression (*r* = 0.012, *P* = 0.942) (Figure 4D).

**Figure 5:**
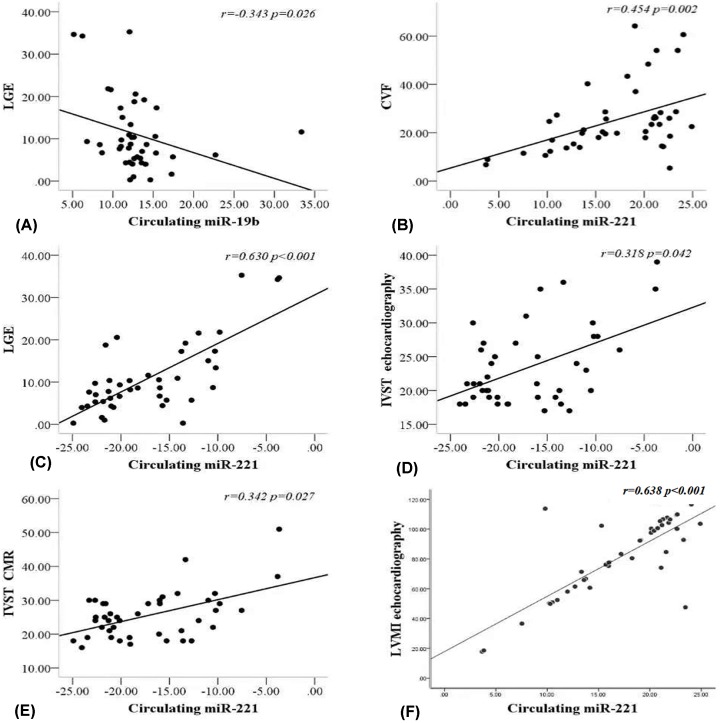
Correlation between circulating miRNA expression and myocardial remodelling parameters (**A**) Negative correlation between mir-19b and LGE (*r* = −0.343, *P* = 0.026). (**B**) Positive correlation between miR-221 expression and CVF (*r* = 0.459, *P* = 0.002). (**C**) Significant positive correlation between miR-221 and LGE (*r* = 0.630, *P* < 0.001). (**D**) MiR-221 expression correlated with MIVST in echocardiogram (*r* = 0.318, *P* = 0.042). (**E**) The level of miR-221 was correlated with MIVST measured by CMR (*r* = 0.342, *P* = 0.027). (**F**) Positive correlation between miR-221 expression and LVMI calculated by echocardiography (*r* = 0638, *P* < 0.001); CVF, collagen volume fraction; IVST, interventricular septal thickness; LGE, late gadolinium enhancement; LVMI, left ventricular mass index. *P* values were for bivariate correlation analysis.

Analysis of the correlation between myocardial remodeling parameters and circulating miRNA expression revealed that (Figure 5): the level of circulating miR-19b expression was significantly and negatively correlated with LGE (*r* = −0.343, *P* = 0.026) (Figure 5A), but not correlated with the maximum interventricular septal thickness (MIVST) of echocardiography (*r* = −0.201, *P* = 0.201) and MIVST of CMR (*r* = −0.135, *P* = 0.396). In addition, the circulating miR-19b was only correlated with the left ventricular mass index (LVMI) of CMR (*r* = 0.214, *P* = 0.046) (Supplementary Figure S2I), and there was no significant correlation with LVMI measured by echocardiography (*r* = 0.130, *P* = 0.225) (Supplementary Figure S2J) and left ventricular ejection fraction (LVEF) (*r* = −0.167, *P* = 0.291) (Supplementary Figure S2K); the circulating miR-221 was significantly correlated not only with CVF (*r* = 0.459, *P* = 0.002) (Figure 5B) and LGE (*r* = 0.630, *P* < 0.001) (Figure 5C), but also with the MIVST measured by echocardiography (*r* = 0.318, *P* = 0.042) (Figure 5D) and CMR (*r* = 0.342, *P* = 0.027) (Figure 5E). Furthermore, circulating miR-221 was significantly correlated with LVMI calculated by echocardiography (*r* = 0.638, *P* < 0.001) (Figure 5F) and CMR (*r* = 0.725, *P* < 0.001) (Supplementary Figure S2G), and it has a negative correlation with LVEF (*r* = 0.557, *P* < 0.001) (Supplementary Figure S2H; Supplementary Figure S2 online).

### Circulating miR-221 has higher value in predicting myocardial fibrosis in HOCM

**Figure 6:**
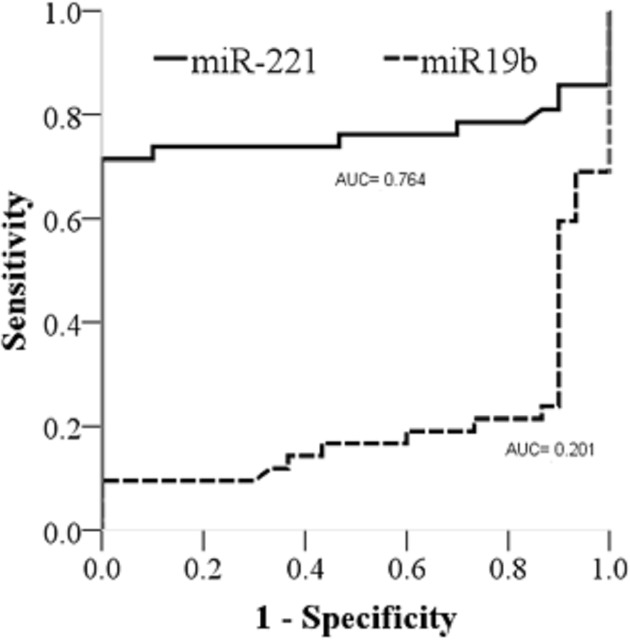
ROC curve of miR221 and miR-19b- in plasma AUC, area under curve; ROC, receiver operating characteristic.

ROC curve analysis, the AUC of miR-221 in plasma was higher than that of miR-19b, and they were 0.764 and 0.201, respectively (Figure 6). The optimal cut-off point, sensitivity, and specificity of miR-221 were 13.64, 71.40%, and 66.67%, respectively. Because the AUC value of miR-19b was less than 0.5, there was no diagnostic value.

## Discussion

We had screened the expression level of 11 fibrosis-related miRs in normal myocardium and patients with HOCM and found five miRs with significant differences between two groups. Three of them were significantly increased, namely, miR-221, miR-222, and miR-433. MiR-155 and miR-19b expression were decreased significantly. Further studies showed that miR-221 and miR-19b expressions were correlated with myocardial fibrosis. Moreover, circulating miR-221 expression was not only correlated with myocardial expression level, but also with myocardial hypertrophy parameters, which had a higher AUC than miR-19b, which had higher diagnostic value for myocardial fibrosis in HOCM.

We focused on screening 11 miRNAs, all of which were reported to be involved in regulating other organ hypertrophy and fibrosis. Katti et al. found that cardiac troponin T has a miR-9 binding site, it may be involved in the regulation of troponin level in the heart to regulate myocardial hypertrophy [16]. MiR-155 promotes the transformation of cardiac fibroblasts into myofibroblasts and participates in myocardial fibrosis induced by angiotensin II [17]. MiR-21 plays an important role in fibrosis of the kidney, liver, lung, and other organs [18–20]. In another study, it was found that miR-21 mediated transforming growth factor (TGF)-β1 by targeting jagged1 and promoted myocardial fibrosis [21]. MiR-15a/b activates the signal of myocardial fibrosis in diabetic cardiomyopathy and accelerates myocardial fibrosis [22]. The difference is that in our study, there is no significant difference in miRNA expression in patients with HOCM, which may be related to the specificity of the disease.

We found that 3 of 11 analysed miRNA levels were significantly increased. It is reported that miR-433 expression is up-regulated in liver and kidney fibrosis [23,24]. Lichan et al. found that the expression of miR-433 was increased in dilated cardiomyopathy. Further studies show that miR-433 can simultaneously inhibit the expression of AZIN1 and JNK1 and activate TGF-β1 pathway, and ERK and p38 kinase, thereby promoting myocardial fibrosis [3]. We found that miR-433 expression was up-regulated, suggesting that miR-433 may also be involved in myocardial fibrosis in HOCM patients, but the specific mechanism needs further investigation. MiR-221/222 comes from the same family. Studies have found that miR-221/222 expression in liver fibrosis is associated with the level of α-smooth muscle actin mRNA and collagen A1 (I), which are the main proteins of fibrosis [25]. Wenjun et al. further revealed that miR-222 modulates liver fibrosis in a murine model of biliary atresia by activating the Akt pathway [26]. In addition, miR-222 regulates abnormal myocardial remodelling through its target homeodomain-interacting protein kinase-1 and homeobox containing-1 [27]. In our study, miR-221/222 expressions were significantly increased, suggesting that they were involved in myocardial remodelling. This is consistent with the mentioned previous studies. Furthermore, we found that two miRNA expressions correlated with myocardial fibrosis significantly. MiR-19b, a member of miR-17-92 cluster, has been widely involved in cardiomyocyte proliferation [28] and myocardial fibrosis [29], miR-19b expression is reduced in pulmonary fibrosis, which is involved in fibrosis through negative regulation of DNA methyltransferase-1 [30]. In hypertensive heart disease, down-regulated miR-19b regulates ventricular fibrosis through negative regulation of adrenoreceptor alpha1a [31]. Jvier et al. [9] found that miR-19b expression was down-regulated in both myocardium and serum in 28 patients with aortic stenosis, and there was a significant correlation between them. Hence, circulating miR-19b may be a biomarker of myocardial collagen cross-linking in aortic stenosis. In our study, miR-19b expression was also down-regulated in the myocardium and plasma of HOCM, suggesting that miR-19b was involved in the myocardial remodelling of the two diseases. However, the difference is that there is no correlation between the two, which may be related to the different types of disease. In addition, Lu et al. reported that circulating miR-19b expression was up-regulated in patients with diffuse myocardial fibrosis in HOCM [12]. This is inconsistent with our findings, which may be related to the specificity of patients and diseases, and all of our patients have focal fibrosis.

We found for the first time that circulating miR-221 was significantly increased in HOCM patients and was significantly correlated with myocardial expression. Furthermore, the increased miR-221 expression was correlated not only with the parameters of myocardial fibrosis, but also with the parameters of myocardial hypertrophy and LVEF, suggesting that miR-221 is involved in the regulation of myocardial hypertrophy, fibrosis, and cardiac function. In addition, the AUC of miR-221 is larger than that of miR-19b; therefore, miR-221 has higher diagnostic value. This is consistent with previous studies. MiR-221 has been proved to regulate cell proliferation, differentiation, and migration by regulating several tumour suppressor genes, including PTEN, p27, p57, and PUMA [32,33]. In a study of pulmonary hypertension, Nie et al. found that up-regulated miR-221 activates β-catenin, leading to pulmonary artery smooth muscle cell proliferation [34]. Other studies have found that increased miR-221 in HOCM regulates cardiac hypertrophy through p27/cyclin-dependent kinase-2/mammalian target of rapamycin axis, thereby exacerbating heart failure [35,36]. In fibrosis, miR-221 can induce renal fibrosis through Ets-1 [37]. A recent study found that abnormal expression of miR-221 regulates myocardial fibrosis by regulating multiple genes, including c-Jun N-terminal kinase 1, TGF- β receptor 1 and receptor 2, and ETS proto-oncogene 1 and then activating TGF-β signalling [38]. The lower miRNA-221/222 levels of the article is inconsistent with our study and may be related to disease is different. In addition, Su et al. reported that miR-221 inhibits autophagy and promotes heart failure by modulating the p27/cdk2/mtor axis [39], which is consistent with our results.

hs-cTnT and NT-proBNP have become important serological markers for myocardial fibrosis and cardiac function in patients with heart disease. We found that both of them were correlated with LGE, but not with LVMI (Supplementary Figure S3 online). In addition, Tatsuya et al. report that hs-cTnT is a direct marker of on-going myocardial fibrosis and that NT-proBNP is a marker of left ventricular overload partially associated with myocardial fibrosis [40].

The preent study has some limitations. First, we found that miRNA expression correlated between myocardium and plasma, but we cannot exclude the effect of other organ or tissue sources on plasma miRNA expression. Further investigation on this topic is needed. Second, due to the unstable expression of U6 and 5s in plasma, we selected cel-miR-39 as the standard control after consulting the relevant literature. We diluted it to 100 pm in strict accordance with the instructions and drew the standard curve. Then, we compared the *C*_t_ value of the target gene with the standard curve for quantitative analysis. It can only be used for relative rather than absolute quantitative analysis. Third, different types of standardised compounds are used in the myocardium (U6) and plasma (cel-miR-39), which may have an effect on the results. However, this method has been commonly used [9]. Fourth, we only screened the expression of 11 miRNAs due to funding and did not analyse their expression profile. Finally, the present study focused on the changes of preoperative expression in patients without further follow-up and no postoperative expression.

We found five abnormal expressions of miRNAs by myocardial expression screening, namely, miR-221, miR-222, miR-433, miR-155, and miR-19b. MiR-19b and miR-221 are associated with fibrosis. Only circulating miR-221 was significantly correlated with myocardial expression, myocardial fibrosis, hypertrophy, and LVEF. ROC curve analysis showed that the circulating miR-221 expression had moderate diagnostic value. Therefore, circulating miR-221 may be a potential biomarker for myocardial hypertrophy and fibrosis in HOCM. The present study is the first to reveal this. For future studies, longer follow-up of patients, monitoring of the changes in miR-221, and providing clinical guidance for the treatment and prognosis of the disease are warranted.

## Supplementary Material

Supplementary Figures S1-S3

## Abbreviations

CVF: collagen volume fraction
HOCM: hypertrophic obstructive cardiomyopathy
LGE: late gadolinium enhancement
LVM: left ventricular mass
MIVST: maximum interventricular septal thickness
