# Supplementary material for: MicroRNA-221 is a potential biomarker of myocardial hypertrophy and fibrosis in hypertrophic obstructive cardiomyopathy
Source: Biosci Rep. 2020 Jan 10;40(1):BSR20191234. doi: 10.1042/BSR20191234 (PMC6954366; doi:10.1042/BSR20191234)
Supplement: Supplementary Figures S1-S3 [file BSR-2019-1234_supp.pdf]

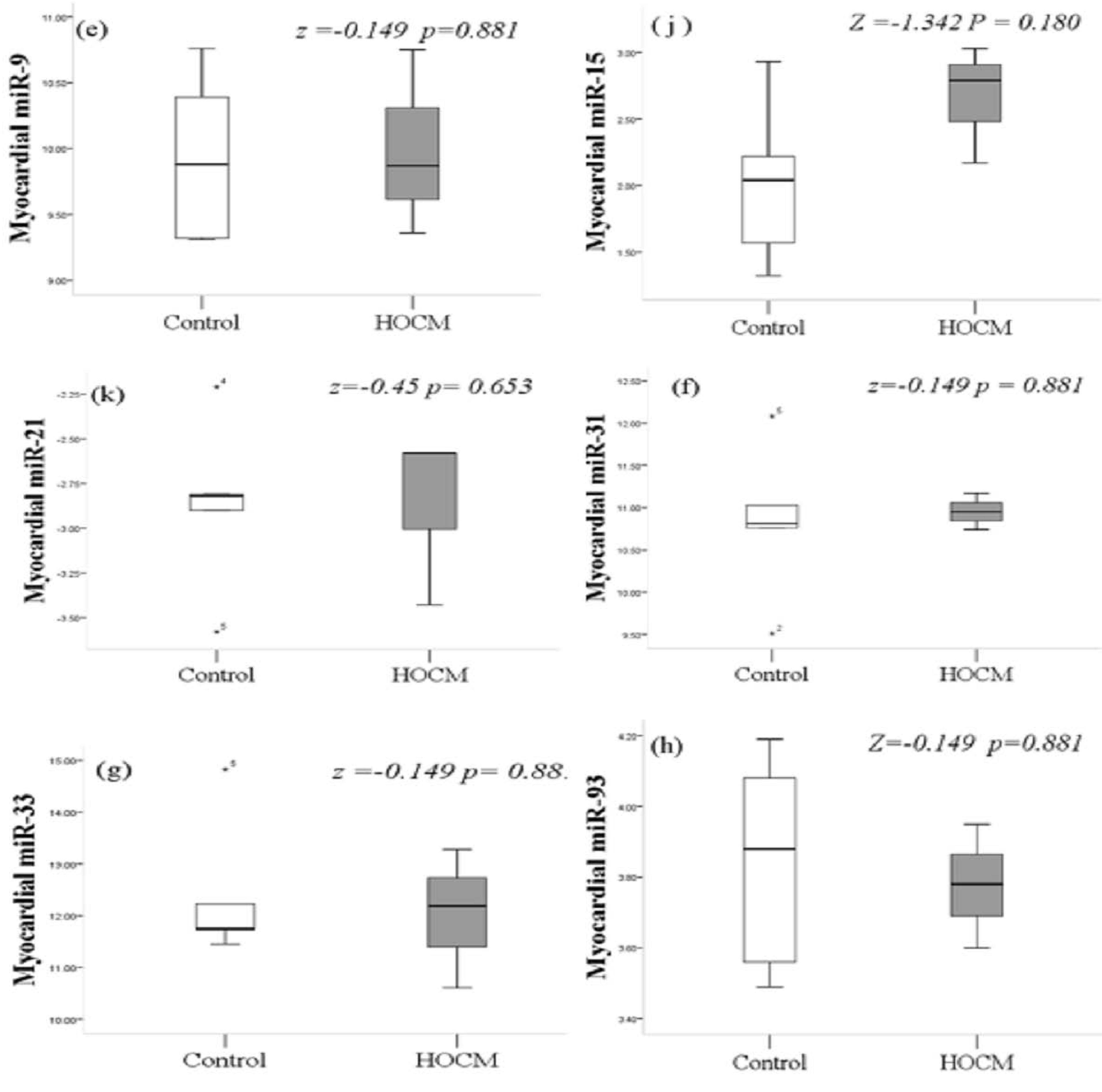
(e) z =-0.149 p=0.881
Myocardial miR-9
Control
HOCM
(j) Z =-1.342 P = 0.180
Myocardial miR-15
Control
HOCM
(k) z=-0.45 p= 0.653
Myocardial miR-21
Control
HOCM
(f) z=-0.149 p = 0.881
Myocardial miR-31
Control
HOCM
(g) z =-0.149 p= 0.88.
Myocardial miR-33
Control
HOCM
(h) Z=-0.149 p=0.881
Myocardial miR-93
Control
HOCM

FigureS1. Six myocardial miRNA expressions in HOCM group were no significantly differences compared with the control group. Namely, miR-9 (e), miR-31(f), miR-33(g), miR-93(h), miR-15 (j) and iR-21 (k). HOCM, hypertrophic obstructive cardiomyopathy

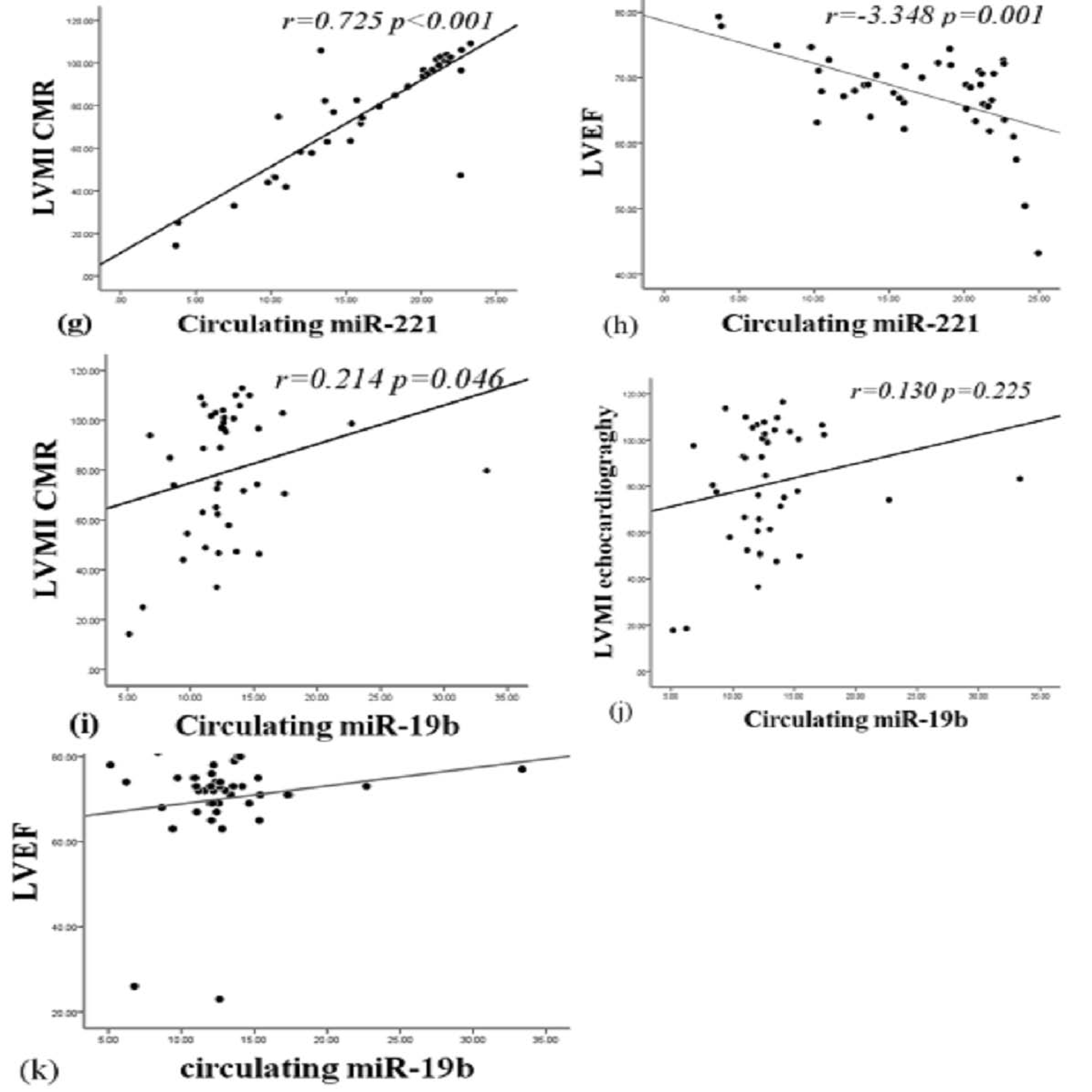
r=0.725 p<0.001
LVMI CMR
(g)
Circulating miR-221
r=-3.348 p=0.001
LVEF
(h)
Circulating miR-221
r=0.214 p=0.046
LVMI CMR
(i)
Circulating miR-19b
r=0.130 p=0.225
LVMI echocardioraghy
(j)
Circulating miR-19b
LVEF
(k)
circulating miR-19b

FigureS2. Correlation between circulating miRNA expression and myocardial remodelling parameters. MiR-221 expression positively correlated with LVMI in CMR (g), negatively correlated with LVEF (h). No correlation between miR-19b and LVMI inCMR (i), LVMI in echocardiography (j) and LVEF (k). CMR means cardiac magnetic resonance, LVMI, left ventricular mass index, LVEF means left ventricular ejection fraction.

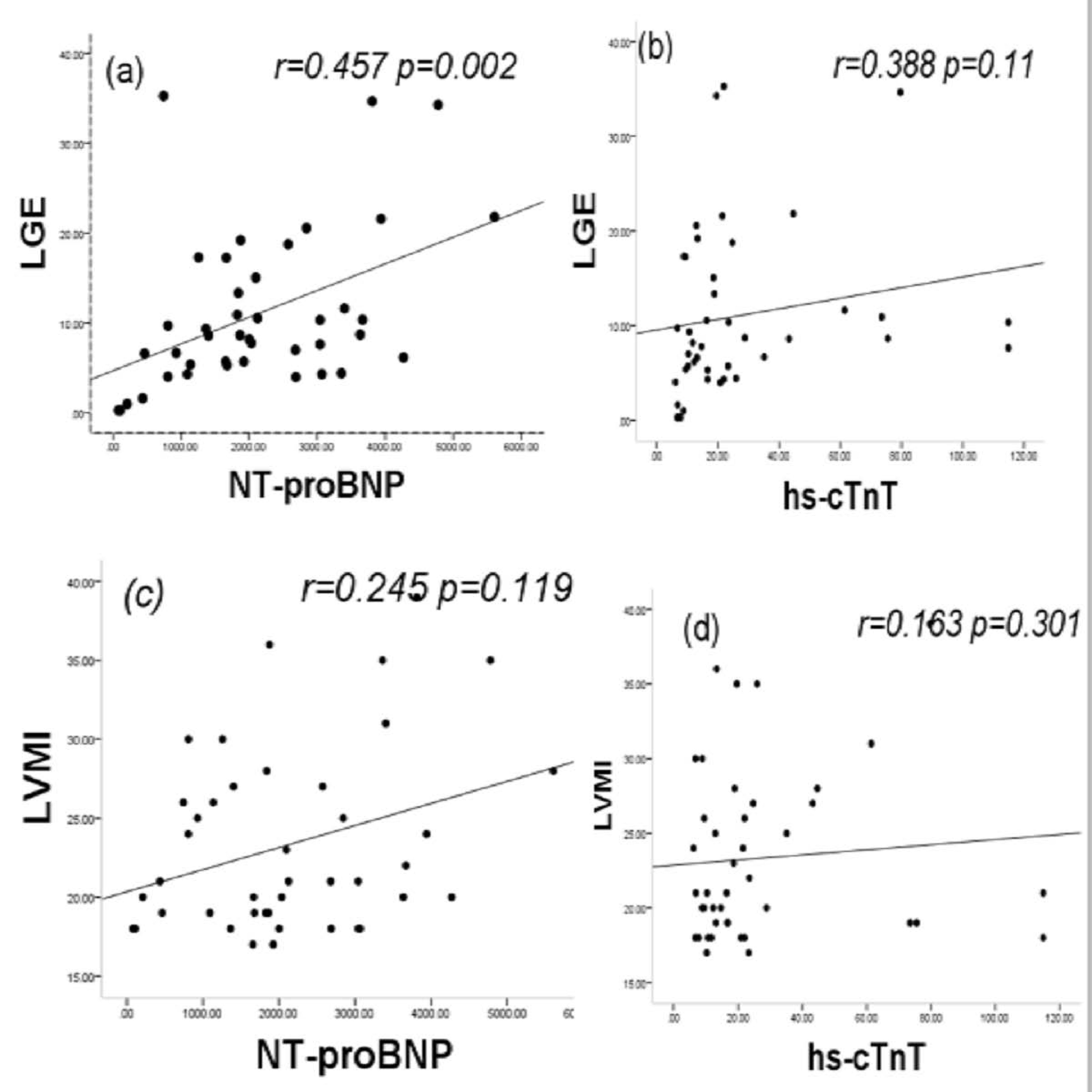

(a)
r=0.457 p=0.002
LGE
NT-proBNP
(b)
r=0.388 p=0.11
LGE
hs-cTnT
(c)
r=0.245 p=0.119
LVMI
NT-proBNP
(d)
r=0.163 p=0.301
LVMI
hs-cTnT

FigureS3.Correlation between NT-proBNP or hs-cTnT and myocardialremodelling.(a) Positive correlation between NT-proBNP and LGE (r=0.457 p=0.002). (b) Positive correlation betweenhs-cTnT and LGE (r=0.388 p=0.011). (c) There was no significant correlation between NT-proBNP and LVMI(r=0.245 p=0.119). (d) There was no correlation between hs-cTnT and LVMI(r=163 p=0.301).LVMI, left ventricular mass index; LGE, late gadolinium enhancement; hs-cTnT, High-sensitivity cardiac troponin T; NT-proBNP, N-terminal pro-brain natriuretic peptide.
